# Pulmonary tumor thrombotic microangiopathy during good response to immuno-chemotherapy for advanced non-small cell lung cancer: a case report

**DOI:** 10.1186/s12890-023-02419-2

**Published:** 2023-04-17

**Authors:** Yoshikazu Utsu, Makio Kawakami, Hironori Arai, Haruka Hisamatsu, Yudai Yano, Jiro Terada

**Affiliations:** 1 Department of Medical Oncology, Japanese Red Cross Narita Hospital, 90-1, Iida-Cho, Narita, 286-8583 Japan; 2 Department of Pathology, Japanese Red Cross Narita Hospital, Narita, Japan; 3 Department of Infectious Disease, Japanese Red Cross Narita Hospital, Narita, Japan; 4 Department of Respiratory Medicine, Japanese Red Cross Narita Hospital, Narita, Japan

**Keywords:** Pulmonary tumor thrombotic microangiopathy, Non-small cell lung cancer, Immune checkpoint inhibitor, Chemotherapy, Case report

## Abstract

**Background:**

Pulmonary tumor thrombotic microangiopathy is a rapidly progressive and fatal disease in which tumor cells embolize to the pulmonary microvasculature. This condition is characterized by severe dyspnea and right heart failure. Although pulmonary tumor thrombotic microangiopathy typically occurs in patients with untreated and/or advanced cancer, its occurrence in patients who are responding well to medical therapy is poorly documented.

**Case presentation:**

A 68-year-old Japanese woman who had received four cycles of immuno-chemotherapy (pembrolizumab, carboplatin, and pemetrexed) followed by three cycles of maintenance therapy (pembrolizumab and pemetrexed) for advanced non-small cell lung cancer and had achieved a partial response with a stable clinical course was admitted to the emergency ward because of worsening breathlessness and general fatigue for 1 week. Chest computed tomography showed no evidence of tumor progression or any new lung lesion. Two-dimensional transthoracic echocardiography demonstrated right atrial and ventricular dilatation, tricuspid regurgitation, and a high trans-tricuspid pressure gradient of 65 mmHg. Despite her percutaneous oxygen saturation being 96% on room air at the time of admission, it worsened rapidly; the patient requiring 8 L/min of oxygen within 4 h. Repeat computed tomography with contrast medium revealed no evidence of pulmonary embolism. The patient developed progressive respiratory failure that was unresponsive to optimal cardio-pulmonary supportive therapy. An autopsy revealed tumorous clusters in pre-capillary lung vessels, whereas the primary lesion had shrunk to the point of almost complete resolution.

**Conclusion:**

Pulmonary tumor thrombotic microangiopathy occurs not only in patients with advanced and/or uncontrolled cancer but also in those whose primary lesion seems to have been well controlled by medical treatment.

## Background

Since von Herbay et al. first reported rapidly progressive and fatal disease involving multiple tumorous thrombi within pulmonary microvasculature in 1990 [1], pulmonary tumor thrombotic microangiopathy (PTTM) has been widely recognized, approximately 200 case reports having been published. Premortem diagnosis of PTTM is challenging because it has a highly aggressive clinical course and lacks specific imaging or laboratory findings; the diagnostic lesions being detectable only by microscopic examination. Although clinicians may suspect PTTM when a patient with untreated and/or advanced cancer presents with dyspnea and has evidence of severe right heart failure, they are less likely to diagnose this condition in patients receiving successful, ongoing medical therapy because this phenomenon has been poorly documented.

Herein, we report our experience of PTTM in a patient with advanced non-small cell lung cancer who had received several cycles of immuno-chemotherapy, achieved a good response, and had apparently well-controlled disease. We have also reviewed relevant published reports.

## Case presentation

A 68-year-old, Japanese, female, ex-smoker (approximately 48 pack-years) was admitted to the emergency ward because of worsening breathlessness and general fatigue for 1 week.

**Fig. 1:**
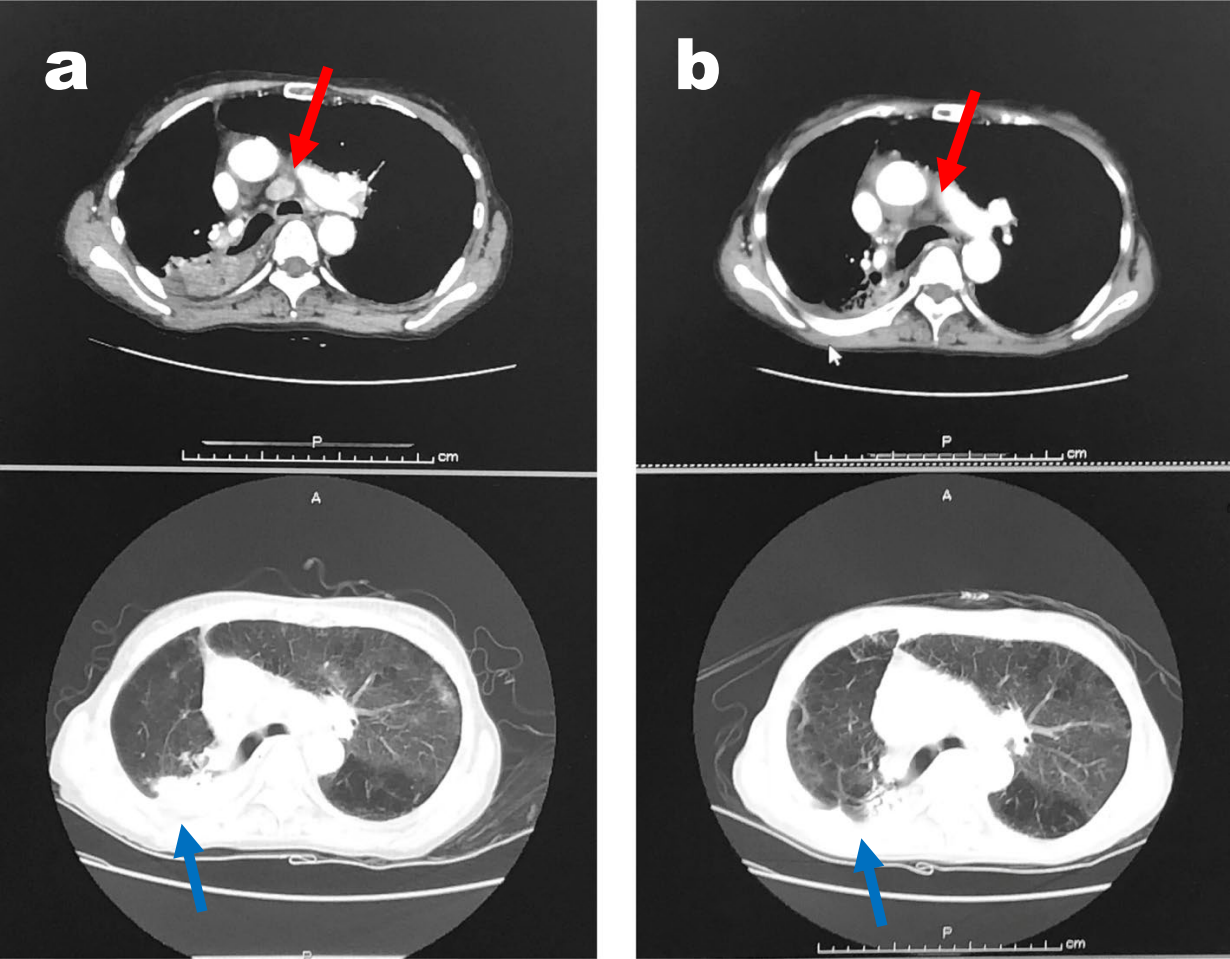
Chest CT scan Chest computed tomography with iodine-based contrast medium. Axial scans: (**a**) prior to, and (**b**) after immuno-chemotherapy. Both involved lymph nodes (red arrows) and the primary lung lesion (blue arrows) are smaller after the immuno-chemotherapy

Five months before this admission, she had been diagnosed with non-resectable advanced non-small cell lung cancer presenting as a 4 cm-diameter right hilar mass with involvement of mediastinal lymph nodes and liver metastasis. Because the tumor was an adenocarcinoma with no PD-L1 expression or targetable gene alteration, she had received four cycles of immuno-chemotherapy (pembrolizumab, carboplatin, and pemetrexed) followed by three cycles of maintenance therapy with pembrolizumab and pemetrexed. She had an uneventful treatment course and a partial response was documented, the primary lesion having shrunk to less than 2 cm in diameter at the last visit (Fig. 1). As instructed, she had not smoked since the diagnosis.

There was no evidence of tumor progression or any other lung lesions on chest computed tomography (CT) performed in the emergency unit. Laboratory testing showed normal liver and renal function, electrolyte balance, and blood cell counts, but a high alkaline phosphatase concentration (1,515 IU/L). Two-dimensional transthoracic echocardiography demonstrated right atrial and ventricular dilatation, tricuspid regurgitation, and a high trans-tricuspid pressure gradient of 65 mmHg. Because right heart strain and pulmonary hypertension were strongly suspected, a repeat chest CT was performed with an iodine-based contrast medium; however, this showed no evidence of embolism within the pulmonary arteries. We assumed that many micro clots in small pulmonary arteries could not be found in the CT scan because of their small size and started anticoagulation therapy including unfractionated heparin. Despite her percutaneous oxygen saturation having been 96% on room air at the time of admission, it worsened so rapidly that she required 8 L/min of oxygen within 4 h. She developed progressive respiratory failure that was unresponsive to optimal cardio-pulmonary supportive therapy and died 20 h after admission.

**Fig. 2:**
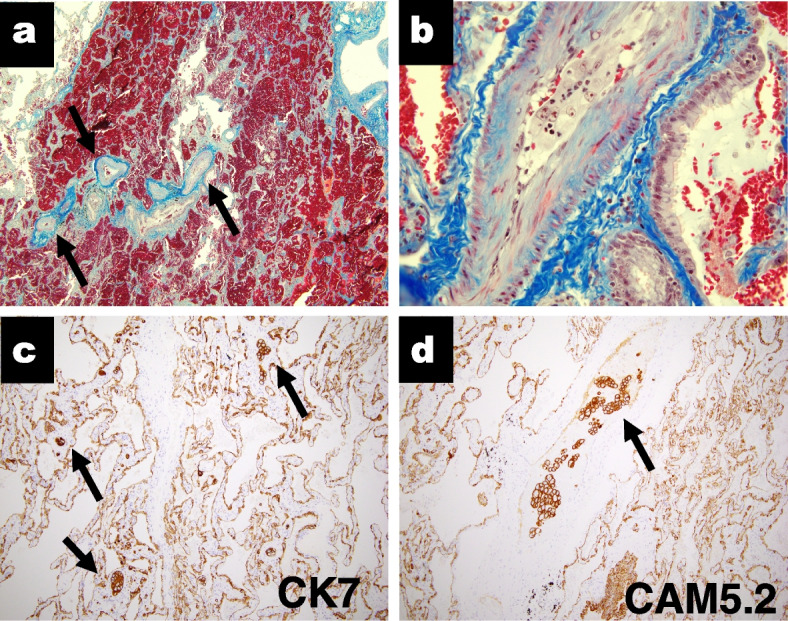
Postmortem pathological findings of the pulmonary vasculature. **a** (Masson-Noguchi staining; magnification × 100) Photomicrograph of pulmonary tissue showing that the lumens of multiple arterioles, veins and lymphatic vessels have been obliterated by tumorous thrombi (arrows). These vessels are surrounded by hemorrhages. **b** (Masson-Noguchi staining; magnification × 400) An arteriole involving fibrocellualr intimal proliferation obliterated by tumor; high-power field. **c** (CK7: Cytokeratin 7; magnification × 100) and (**d**) (CAM5.2: Cytokeratin CAM 5.2; magnification × 100) Homology between the tumorous thrombi in the pulmonary microvasculature and the previously diagnosed lung cancer was confirmed by immunostaining

**Fig. 3:**
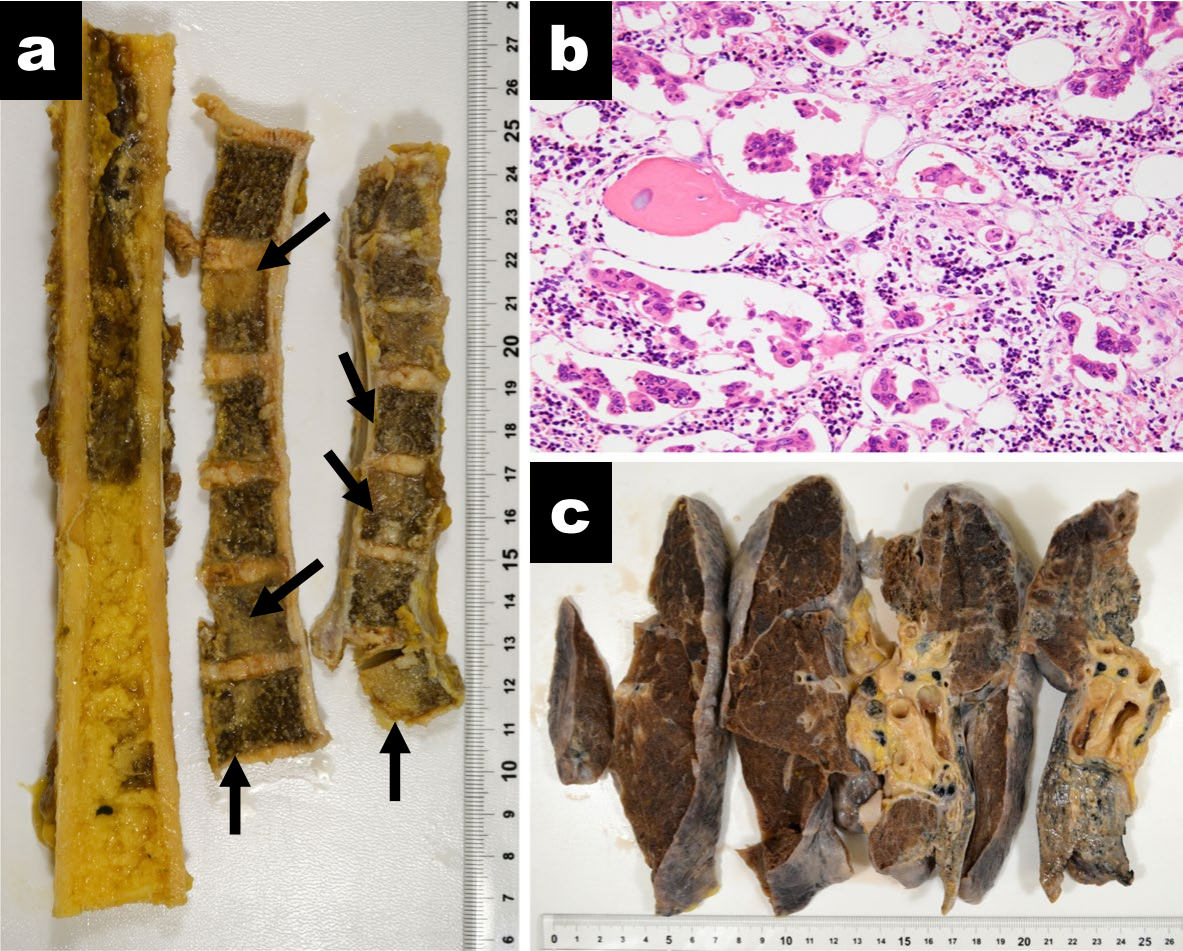
Bone metastasis and Gross pathology of the lung. **a** Multiple metastasis (arrows) in the femur and multiple vertebrae. **b** Bone metastasis; (hematoxylin–eosin staining; magnification × 200); high-power field. **c** Gross pathology of the patient’s right lung. Although there is atelectasis of the lower lobe, no tumors are evident

Postmortem pathological examination revealed tumorous clusters in pre-capillary lung vessels (Fig. 2), evidence of severe right heart strain, including a dilated right atrium and ventricle, severely increased central blood volume (725 mL), and congestion of the liver, resulting in a definitive diagnosis of PTTM. The autopsy also revealed multiple vertebral bone metastases but no tumor re-growth in the primary lesion in the right hilum or mediastinal lymph nodes (Fig. 3); only scattered tumor cells being identified. The liver metastases had resolved. No tumors were found in any other location.

## Discussion and conclusions

Our patient’s primary lung cancer and involved regional lymph nodes responded to treatment and seemed to be well controlled. However, the multiple bone metastases and PTTM that were diagnosed by postmortem examination indicated that the disease had been progressing in other locations. Some case series of autopsies have reported that PTTM typically occurs in patients with advanced metastatic cancer, particularly adenocarcinoma and that its incidence is 0.9%–3.3% [1–4]. Godbole et al. comprehensively reviewed 160 reported case of PTTM and found that PTTM was diagnosed prior to or simultaneously with diagnosis of the causative cancer in 35% of cases [5]. Thus, PTTM can cause collapse and sudden death before the causative cancer has been recognized, indicating that the cited 35% should be viewed with caution as there may be publication biases. Additionally, cases of PTTM in patients receiving immuno-chemotherapy, including immune checkpoint inhibitors, are poorly documented. We believed that our patient’s lung cancer had been successfully treated until the postmortem examination was performed. Such overconfidence may hinder making a premortem diagnosis of PTTM.

Regarding our patient’s clinical course, there was a discrepancy in efficacy of treatment between the primary lung cancer and the bone metastases and tumors in the pulmonary microvasculature. Differences in efficacy in different sites are not rare in clinical practice. Two theories to explain this phenomenon have been proposed. One is the classical hypothesis of Goldie and Coldman that drug resistance can arise from spontaneous gene mutations and the risk of such resistance increases with time [6]. The other is based on the fact that whole-genome sequencing of metastases and primary tumors has recently revealed that polyclonal seeding and inter-clonal cooperation between multiple subclones occurs, potentially generating differences in biological properties between metastases and primary tumors [7]. In the present case, the differences in treatment efficacy may be attributable to selective migration of surviving and resistant clones to the vertebrae and pulmonary microvasculature. Additionally, reducing the primary tumor may be responsible for exacerbation of metastatic disease: Demicheli et al. reviewed several cases in which explosive metastatic growth was observed soon after resection of the primary tumor. They hypothesized that the primary tumor releases systemic factors that suppress growth of micrometastases and that resection of a primary cancer can modulate tumor dormancy [8]. Our patient’s primary tumor was not resected. However, she did receive potent immuno-chemotherapy, including immune checkpoint inhibitors, which shrank her primary tumor, potentially reducing release of systemic suppressive factors in the same way as after resection.

Making a premortem diagnosis of PTTM is extremely difficult because it is rare and progresses rapidly. Furthermore, a bronchoscopic or thoracoscopic biopsy, which is a high-risk procedure in such ill patients, is needed for a definitive diagnosis. Several attempts at making an early diagnosis of PTTM by less invasive means have been reported. Kamada et al. reported identifying specific findings for PTTM in three patients by lung perfusion blood volume imaging on dual-energy computed tomography [9]. Mitsui et al. suggested cytological examination of blood sampled via a right heart catheter [10], previous studies having shown that cytological examination of blood obtained via a wedged pulmonary artery catheter has a sensitivity of 80%–88% and a specificity of 82%–94% [11, 12]. These approaches are promising and could enable more timely treatment; however, they can only be implemented if PTTM is suspected.

In conclusion, clinicians should always consider the possibility of PTTM when a patient with diagnosed or suspected cancer develops progressive dyspnea, because PTTM must be suspected before it can be diagnosed.

## Data Availability

All data generated or analyzed during this study are included in this published article.

## Abbreviations

CAM5.2: Cytokeratin CAM 5.2
CK7: Cytokeratin 7
CT: Computed tomography
PTTM: Pulmonary tumor thrombotic microangiopathy
